# Effect of Cr on Aqueous and Atmospheric Corrosion of Automotive Carbon Steel

**DOI:** 10.3390/ma14092444

**Published:** 2021-05-08

**Authors:** Sang-won Cho, Sang-Jin Ko, Jin-Seok Yoo, Yun-Ha Yoo, Yon-Kyun Song, Jung-Gu Kim

**Affiliations:** 1 Department of Materials Science and Engineering, Sungkyunkwan University, 2066 Seobu-Ro, Jangan-Gu, Suwon-Si 16419, Korea; jsw2811@gmail.com (S.-w.C.); tkdwls121@skku.edu (S.-J.K.); wlstjr5619@skku.edu (J.-S.Y.); 2 POSCO Global R&D Center, Steel Solution Research Laboratory, 100 Songdogwahak-Ro, Yeonsu-Gu, Incheon 21985, Korea; yunha778@posco.co.kr (Y.-H.Y.); petersong@posco.com (Y.-K.S.)

**Keywords:** automotive steel, atmospheric corrosion, electrochemical impedance spectroscopy, cyclic corrosion test, iron oxide

## Abstract

This study investigated the effect of Cr alloying element on the corrosion properties of automotive carbon steel (0.1C, 0.5Si, 2.5Mn, Fe Bal., composition given in wt.%) in aqueous and atmospheric conditions using electrochemical measurement and cyclic corrosion tests. Three steels with 0, 0.3, and 0.5 wt.% Cr were studied by electrochemical impedance spectroscopy. Polarization resistance (R_p_) of 0.3 Cr and 0.5 Cr steels was higher than that of 0 Cr steel, and the R_p_ also increased as the Cr content increased. Therefore, Cr increases the corrosion resistance of automotive carbon steel immersed in a chloride ion (Cl^−^)-containing aqueous solution. In the cyclic corrosion test results, Cl^−^ was concentrated at the metal/rust interface in all of the steels regardless of Cr content. The Cl^−^ was uniformly concentrated and distributed on the 0 Cr steel, but locally and non-uniformly concentrated on the Cr-added steels. The inner rust layer consisted of β-FeOOH containing Cl^−^ and Cr-goethite, while the outer rust layer was composed of amorphous iron oxyhydroxide mixed with various types of rust. FeCl_2_ and CrCl_3_ are formed from the Cl^−^ nest developed in the early stage, and the pitting at CrCl_3_-formed regions are locally accelerated because Cr is strongly hydrolyzed to a very low pH.

## 1. Introduction

Steel sheets for automobiles are exposed to various corrosive environments due to climate change. In particular, the increased inundation from heavy rain and the use of salt for snow removal accelerate the corrosion of the automobile steel sheet, which leads to the deterioration of the durability and the collision safety of the vehicle [1]. Furthermore, with the recent rapid development of industry, these corrosive environments are becoming more and more severe. Therefore, it is essential to evaluate the corrosion life of the steel for predicting the durability of automobile parts. Currently, corrosion life is most often evaluated by the salt spray test (SST) and cyclic corrosion test (CCT). The accelerated CCT method for simulating an actual environment is used by many automakers. When an automotive carbon steel (ACS) sheet is evaluated through a CCT, atmospheric corrosion occurs on the test specimen and is greatly affected by environmental factors such as the type of material, humidity, time of wetness (TOW), and temperature [2]. For example, in the case of Cu and Ag, the corrosion rate is most affected by sulfides such as H_2_S and SO_2_ in the air, whereas in the case of Fe, acid fumes and fine dust are known to be more important. Furthermore, in coastal cities, the corrosion rate changes depending on the chloride concentration in the air. When the salt particles in the air are adsorbed on the metal surface, water may be condensed on the surface even if the relative humidity is low, which facilitates the formation of a water film [3]. As the TOW lengthens, the corrosion rate increases.

Generally, weathering steel is widely used to prevent atmospheric corrosion and contains alloying elements such as Cr, Cu, and Ni. Particularly, Cr is known as an element that improves the corrosion resistance of low alloy steel in various corrosive environments. When the weathering steel is exposed to a corrosive environment, Fe oxides (porous rust layer) are initially formed under attack from oxygen, similarly to common steel. However, over time, a dense rust layer (protective rust) is formed on the steel surface. This rust layer protects the steel surface, inhibiting corrosion and reducing corrosion rates compared to common steel. In common steel, the oxide rust layer penetrates into the substrate, and corrosion of the substrate continues, while in weathering steel, the amorphous layer enriched with Cu, Cr, and Ni inhibits further corrosion progress. Weathering steel forms a thin rust layer with FeOOH as the main component on the steel surface in the early stages. Then, a Cr-enriched layer (Cr-goethite) with very small particles containing Cr is formed on the steel surface [4,5]. The Cr-goethite layer has cation selectivity, preventing the penetration of corrosion substances such as SO_4_^2^^−^ and Cl^−^ from the outside [6,7,8]. However, unlike these positive effects, negative effects have also been reported. According to Park et al. [9], in the flue gas desulfurization environment, Cr induces localized corrosion when Cr and Cu are added together in low-carbon steel because Cr segregates into the grain boundary, forming a Cr depletion region.

As described above, many research endeavors have been undertaken with respect to Cr’s effect on the corrosion of metallic materials; however, only a few studies on the effect of Cr on the corrosion of ACS have been conducted. In this study, the effect of the Cr alloying element on the aqueous corrosion and atmospheric corrosion of ACS was investigated. The aqueous corrosion properties were analyzed using electrochemical measurements in a chloride (Cl^−^)-containing solution, and atmospheric corrosion properties were analyzed via a CCT.

## 2. Materials and Methods

**Figure 1:**
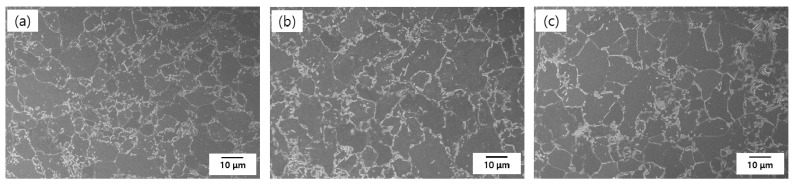
SEM images of the microstructure of (**a**) 0 Cr, (**b**) 0.3 Cr, and (**c**) 0.5 Cr, etched with 2% nital solution.

The specimens used in the electrochemical test and CCT were ACS containing 0, 0.3, and 0.5 wt.% Cr (produced by POSCO, Gwangyang, Korea), as described in Table 1. The microstructure images of the specimens are shown in Figure 1. In Figure 1, all the steels are composed of ferrite and martensite phases. Most martensite was formed along the grain boundary, with a small amount present inside the ferrite matrix. There is no noticeable difference in the three steels except for the grain size. The higher the Cr content, the bigger the grain size.

The specimens were cut to a size of 1.5 cm × 1.5 cm, polished with 600-grit SiC paper, and cleaned with distilled water. Additionally, the solution for electrochemical measurements was 3.5 wt.% NaCl. All of the electrochemical measurements were conducted in a 3-electrode electrochemical cell. The test specimen was used as the working electrode, a carbon rod was used as the counter electrode, and a saturated calomel electrode was used as the reference electrode. Potentiodynamic polarization tests were performed with a potential sweep of 0.166 mV/s according to ASTM G5. To establish a stable potential, the scan was initiated after the specimen was stabilized in the solution [10]. Electrochemical impedance spectroscopy (EIS) tests were performed with an amplitude of 10 mV in the frequency range of 100 kHz to 10 mHz. Electrochemical tests were conducted by a potentiostat (BioLogics, VMP-2, Seyssinet-Pariset, France). After the CCT, the test specimens were mounted with epoxy and analyzed by an optical microscope (OM), and the components of the corrosion product were analyzed by an electron probe micro-analyzer (EPMA; JEOL, JXA-8530F, Fukuoka, Japan), X-ray diffraction (XRD; Rigaku, D/max-2500V/PC, Tokyo, Japan), and transmission electron microscopy (TEM; FEI, Tecnai F20 G2, Hillsboro, OR, USA).

**Figure 2:**
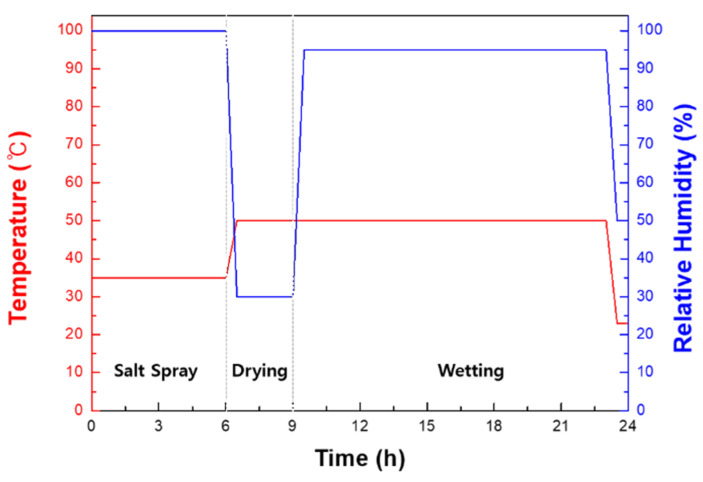
Specific conditions for the cyclic corrosion test.

The specific CCT process is shown in Figure 2. The specimens used for the CCT were cut to a size of 3 cm × 7 cm, and exposed on only one side to the corrosive environment. The CCT was performed for 10, 20, and 30 cycles, respectively. The salt solution used for the CCT was 5 wt.% NaCl. The length of a CCT cycle was 24 h, consisting of a wet stage for 21 h and a dry stage at 30% of relative humidity and 50 °C for 3 h.

## 3. Results and Discussion

### 3.1. Electrochemical Measurement

**Figure 3:**
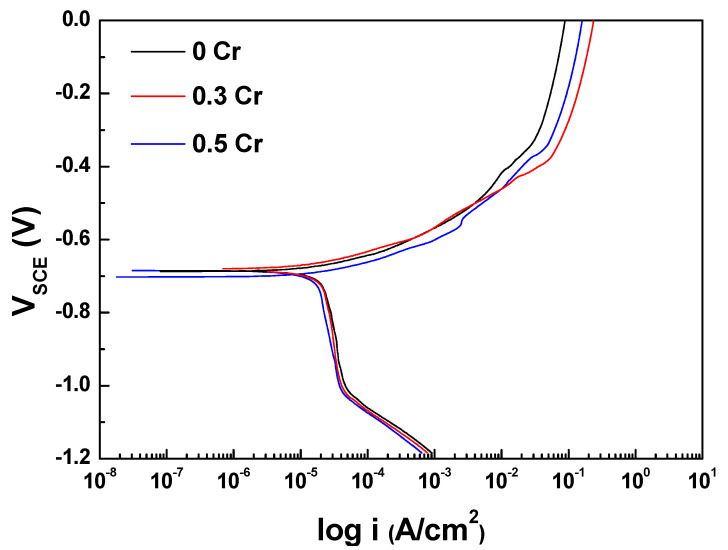
Potentiodynamic polarization curves in 3.5 wt.% NaCl solution.

Potentiodynamic polarization tests were conducted to analyze the difference in corrosion characteristics depending on the Cr content, and the results are shown in Figure 3 and Table 2. In Figure 3, all of the specimens show an active corrosion behavior that increases with increasing potential in Cl^−^-containing environments without passivation. Additionally, there was no significant difference in the corrosion potential regardless of the Cr content. The corrosion potential (E_corr_) and corrosion current density of 0.5 Cr steel were slightly lower than that of 0 Cr and 0.3 Cr steels, but this is an insignificant difference that can be regarded as an experimental error.

**Figure 4:**
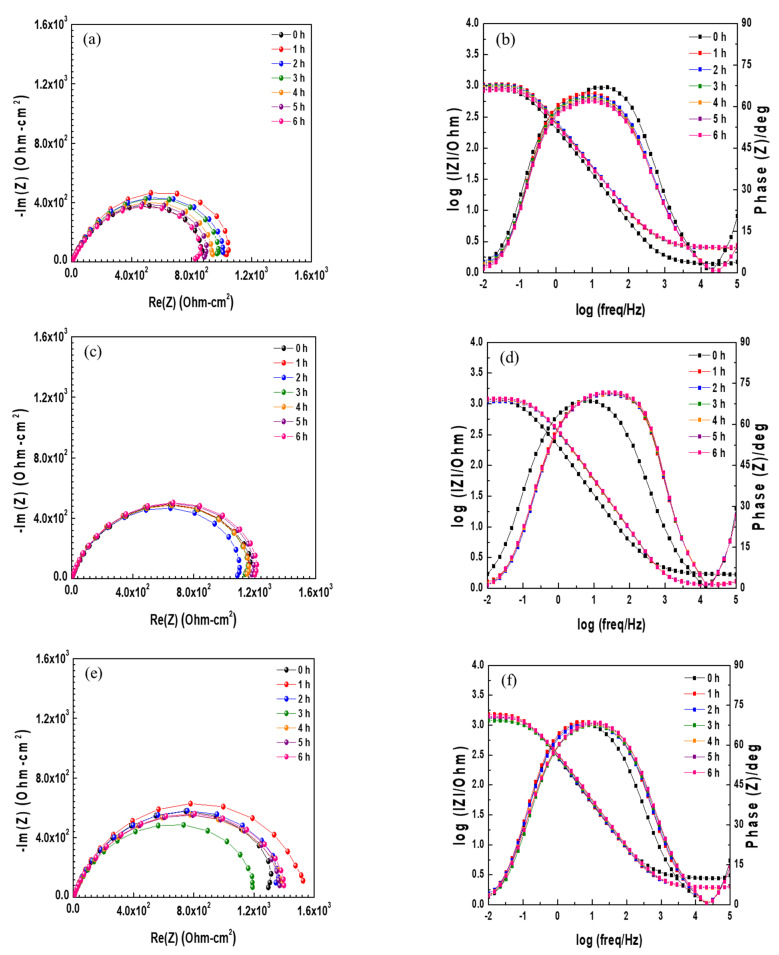
Nyquist and Bode impedance plots of EIS data of (**a**,**b**) 0 Cr steel, (**c**,**d**) 0.3 Cr steel, and (**e**,**f**) 0.5 Cr steel in 3.5 wt.% NaCl solution.

In order to obtain a better understanding of the effect of Cr on the corrosion behavior of ACS under aqueous conditions, EIS measurement was performed in a 3.5 wt.% NaCl solution at room temperature. Figure 4 shows the results of EIS measurement in the form of Nyquist and Bode plots under open circuit potential (OCP) according to various immersion times. The Nyquist plots were not perfect semicircles due to dispersion effects that are often caused by the geometrical inhomogeneity or non-uniform current distribution on the electrode surface [11]. The capacitive loops in the high- and low-frequency regions overlapped. The capacitive loop of the high-frequency region showed the resistance of the film, whereas that of the low-frequency region showed the charge transfer resistance [12,13,14]. In the 0 Cr steel, the size of the semicircle for 1 h was larger than that for 0 h, and then became smaller with respect to immersion time. This means that a thin and weak oxide layer was formed on the steel surface at the initial stage of the corrosion process, and deteriorated with respect to immersion time due to its instability [15]. Similar to 0 Cr steel, the size of the semicircles for the 0.3 Cr and 0.5 Cr steels also increased immediately after immersion, and then decreased with immersion time. However, the size of the overall capacitive semicircles is ordered as 0.5 Cr > 0.3 Cr > 0 Cr. In general, the size of the capacitive semicircle on the Nyquist plot represents corrosion resistance. This means that the Cr alloying element within 0.5 wt.% improves corrosion resistance in an aqueous environment. In the Bode plots, the impedance at a low frequency and the shoulder width at the phase angle were increased and wider immediately after immersion, and then decreased and narrower with immersion time. This result is consistent with the Nyquist impedance interpretation.

**Figure 5:**
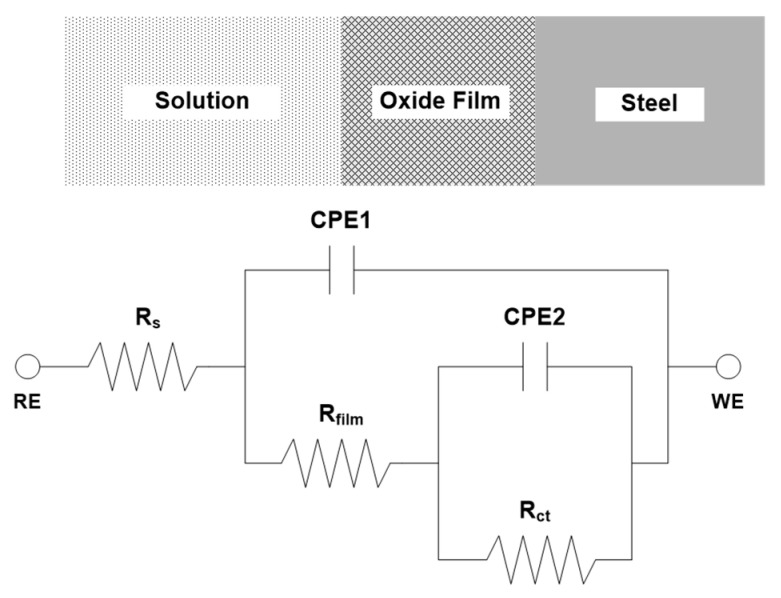
Equivalent circuit for ACS in 3.5 wt.% NaCl solution.

To determine the optimized values for the resistance and capacitance parameters, the equivalent circuit was used as shown in Figure 5. R_s_ is the test solution resistance, R_film_ is the oxide film resistance, R_ct_ is the charge transfer resistance, and R_film_ + R_ct_ is total resistance or polarization resistance (R_p_), which is proportional to the radius of the capacitive loop in the Nyquist plot. The constant phase element (CPE) is the capacitive response of the system. CPE1 is the capacitive response of the oxide film, and CPE2 is the capacitive response of the double layer caused by the dissolution of the metal and the charge separation between the metal/electrolyte interface [16,17,18,19]. In the equivalent circuit, CPE is defined as below:

(1) $$Z_{\mathrm{CPE}}={\;Q}_{0}^{-1}{\left(j\omega \right)}^{-n}$$

where Z is the impedance, Q_0_ is the coefficient of proportionality, j is the imaginary number, ω is the angular frequency, and n is the empirical CPE exponent (0 ≤ n ≤ 1) measuring the deviation from the behavior of an ideal electric capacity [20,21]. CPE can represent resistance (n = 0), capacitance (n = 1), inductance (n = −1), or Warburg impedance (n = 0.5) in accordance with n [22]. 

The EIS data were fitted using the ZSimpWin (Princeton Applied Research, Oak Ridge, TN, USA) program and the results are shown in Table 3. The R_p_ of 0.3 Cr and 0.5 Cr steels was higher than that of 0 Cr steel, and the R_p_ also increased as the Cr content increased. This indicates that the corrosion resistance is increased as the Cr content increases. This is believed to be due to the bigger grain size of the steel with higher Cr content. Metal with active polarization behavior decreases the corrosion rate with bigger grain size [23]. Therefore, Cr improved the corrosion resistance of the ACS that was immersed in the Cl^-^-containing aqueous solution.

### 3.2. Cyclic Corrosion Test Results

**Figure 6:**
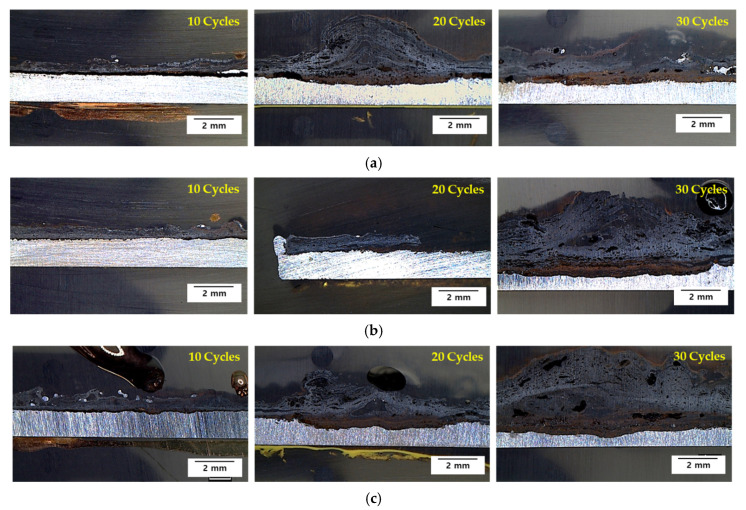
Cross-section OM images of (**a**) 0 Cr steel, (**b**) 0.3 Cr steel, and (**c**) 0.5 Cr steel after CCT.

The specimens after the CCT were cut and the cross-section was observed with an OM and the results are shown in Figure 6. After 10 cycles, the rust of all steels was thin and relatively uniform. However, after 20 cycles, brown and black oxides were formed on the inner layer and outer layer, respectively. Especially after 30 cycles, the amount of corrosion product of 0.3 Cr and 0.5 Cr steels was greater than that of 0 Cr steel, and corroded in a more localized way. Furthermore, since the thickness of the oxide layer is proportional to the amount of corrosion of the base metal, a thick oxide layer was locally formed on the 0.3 Cr and 0.5 Cr steels.

**Figure 7:**
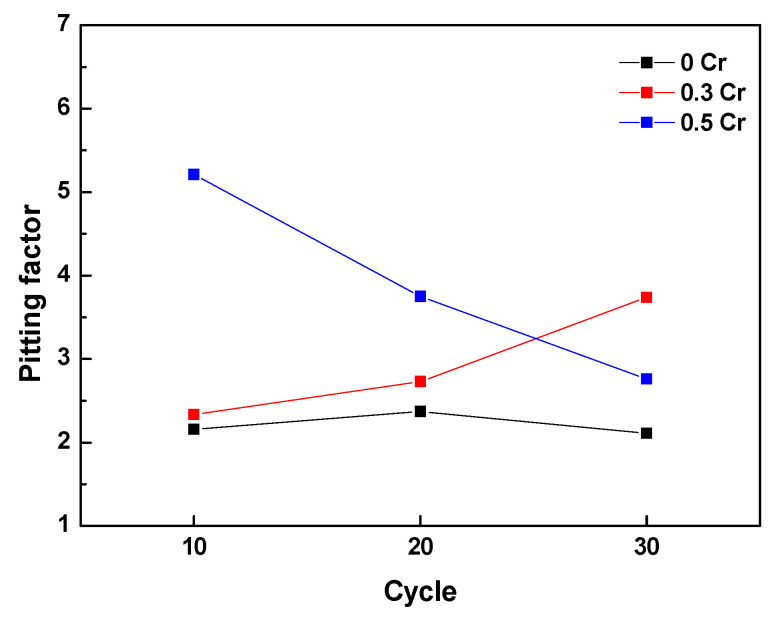
Variation of pitting factor according to CCT cycle.

To determine the localized corrosion tendency, the pitting factor (PF) with a concept similar to that given in ASTM G46 was used. A PF value of 1 means perfect uniform corrosion, and a higher PF means an increased localized corrosion tendency. The PF for each CCT cycle was derived by the following equation, and the variation of the PF according to CCT cycle is shown in Figure 7.

(2) $$\mathrm{PF}=\frac{p}{d}$$

where p is the maximum penetration depth, and d is the average penetration depth.

In the case of 0 Cr steel, the PF was approximately 2 regardless of the cycle, while the PF of 0.3 Cr and 0.5 Cr steels changed depending on the cycle. In all cycles, the PF of the Cr-added steels was higher than that of the 0 Cr steel, but the PF was not proportional to Cr content. This indicates that the Cr alloying element can accelerate localized corrosion, and the presence or absence of Cr greatly affects the localized corrosion, not the Cr content.

**Figure 8:**
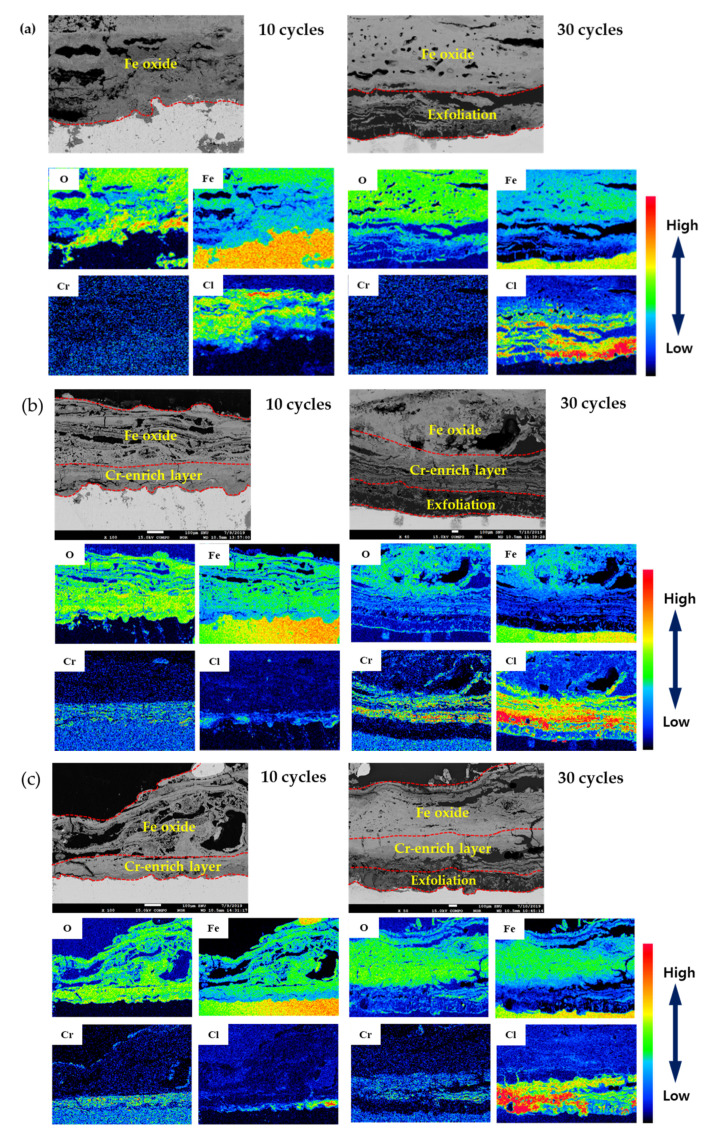
EPMA analysis of (**a**) 0 Cr steel, (**b**) 0.3 Cr steel, and (**c**) 0.5 Cr steel after CCT.

The cross-section of the specimen after 10 and 30 cycles was analyzed to determine the chemical composition using EPMA, and the results are shown in Figure 8. The rust layer of 0 Cr steel was composed entirely of porous iron oxide (e.g., γ-FeOOH, γ-Fe_2_O_3_, Fe_3_O_4_). In addition, Cl^−^ was accumulated at the metal/rust interface and on the inner layer with uniform concentration and distribution. The Cr-added steels had a very dense and uniform Cr-enriched region in the inner rust layer, while the outer rust layer was composed of porous iron oxide, like 0 Cr. Cl^−^ was detected underneath the Cr-enriched layer and at the metal/rust interface, but unlike 0 Cr steel, it was localized and non-uniformly concentrated. The rust layer of the 30-cycle steel was exfoliated from the metal, and the Cl^−^ concentration in the inner rust layer was increased significantly compared to 10 cycles. Therefore, it is considered that the corrosion is accelerated because the protective oxide layer loses its protective property after the rust layer exfoliates.

To summarize the above results, Cl^−^ was concentrated at the metal/rust interface in all of the specimens regardless of Cr content. Generally, since the localized corrosion in an atmospheric environment is caused by Cl^−^ enrichment [4,24], localized corrosion with a PF of approximately 2 or higher occurred in all of the steels, as shown in Figure 7. However, 0.3 Cr and 0.5 Cr steels had higher PFs than 0 Cr steel because Cl^-^ was localized and non-uniformly concentrated as compared with 0 Cr steel.

### 3.3. Rust Constituent Analysis

**Figure 9:**
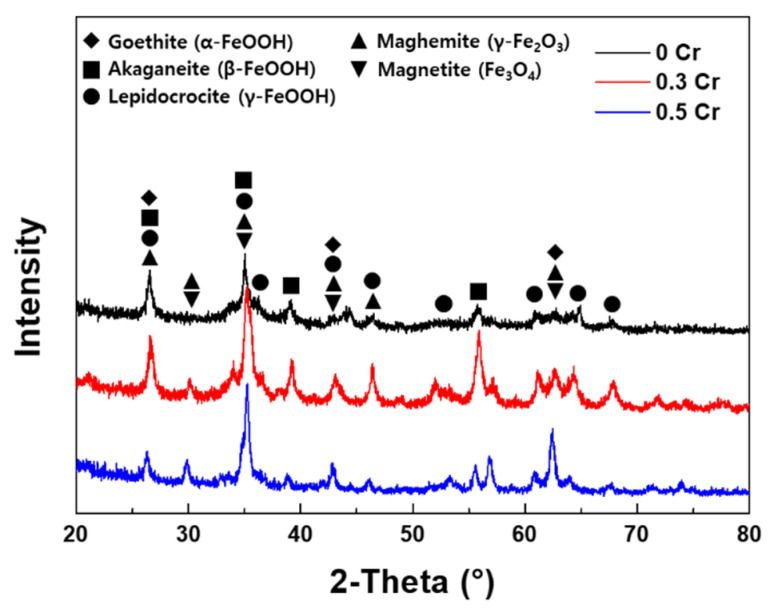
XRD analysis of the specimen surfaces after 20 cycles of CCT.

**Figure 10:**
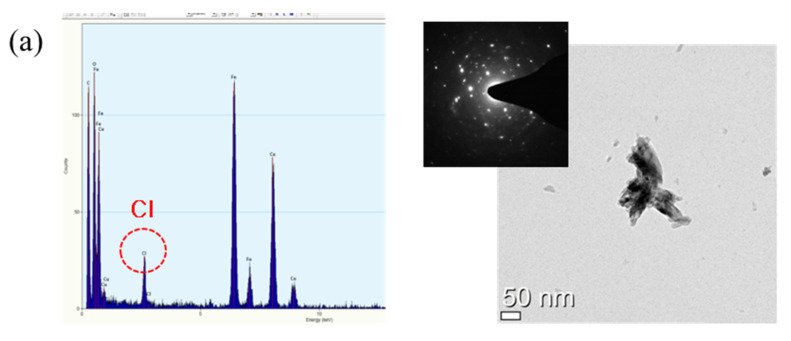

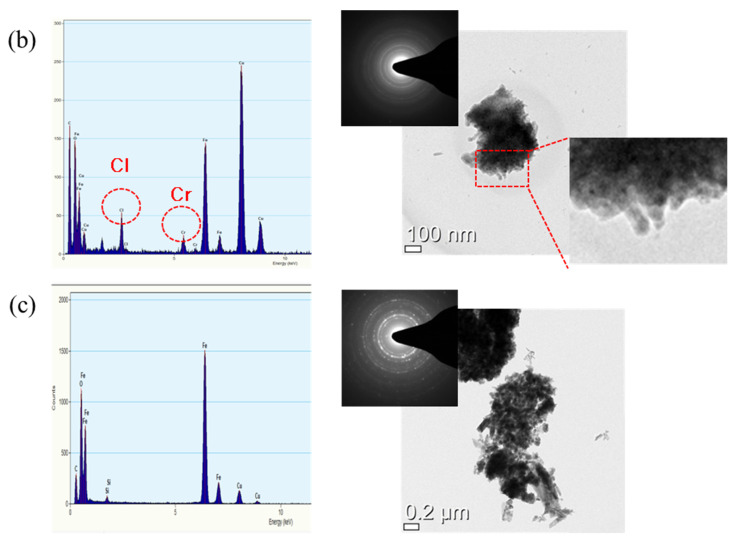
TEM images and the diffraction patterns of (**a**,**b**) inner rust and (**c**) outer rust formed on the 0.5 Cr steel.

The constituent of the rust formed after the CCT was analyzed by XRD, EDS, and TEM, and the results are shown in Figure 9 and Figure 10. As shown in Figure 9, the phase of rust formed by the CCT was almost the same for all steels. The rusts were composed of various oxides and hydroxides such as α-FeOOH (goethite), β-FeOOH (akaganeite), γ-FeOOH (lepidocrocite), γ-Fe_2_O_3_ (maghemite), and Fe_3_O_4_ (magnetite). Among them, akaganeite always contains Cl^−^ in the lattice because it is stabilized by the Cl^−^ entering the lattice structure. In addition, akaganeite is formed only during the dry stage in an atmospheric environment, and Cl^−^ in the akaganeite is dissolved in water to promote corrosion during the wet stage. That is, the akaganeite acts as a Cl^−^ reservoir. As a result, a large amount of akaganeite is formed inside the pit generated by atmospheric corrosion [3,6,24,25,26]. Therefore, akaganeite was formed in the inner rust layer, and Cl^−^ was observed at the metal/rust interface, as shown in Figure 8.

The EDS results, TEM images, and diffraction patterns of the inner and outer rust formed on the 0.5 Cr steel were analyzed, and the results are shown in Figure 10. Chlorine was observed in the inner rust particle as an acicular single crystal with a size of about 100 nm, as shown in Figure 10a. This is the major characteristic of akaganeite [27]. In Figure 10b, Cr and Cl^−^ were observed together in the inner rust particle. The particle was polycrystalline and was a spherical agglomeration with a size of several nanometers. As the spherical-shaped rust is the main feature of goethite [28], the particle is Cr-containing nanoscale goethite (Cr-goethite). Since dissolved or enriched Cr suppresses the growth of goethite crystals [7], the size of the Cr-goethite particles is very small. Cr-goethite is so small in size that it acts as a protective film that is densely formed in the inner rust layer. Furthermore, Cr-goethite has cation selectivity so it can inhibit the penetration of aggressive anions such as Cl^−^ and SO_4_^2−^ and improve corrosion resistance [7,25,29,30,31]. In short, Cr-goethite was formed in the inner rust layer of Cr-added steels, which blocked the inflow of additional Cl^−^ from the outside and consequently improved the corrosion resistance. As shown in Figure 10c, Cl^−^ and Cr were not detected in the outer rust particle. Therefore, the outer rust layer is composed of various rusts such as lepidocrocite, maghemite, and magnetite detected from the XRD analysis results. In summary, the inner rust layer consists of akaganeite containing Cl^−^ and Cr-goethite, while the outer rust layer is composed of amorphous iron oxyhydroxide mixed with various types of rust.

### 3.4. Localized Corrosion Mechanism of Cr-Added Steel under Wet/Dry Conditions

**Figure 11:**
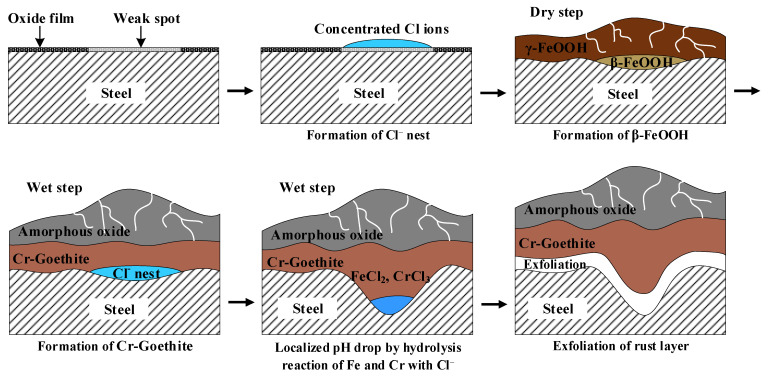
Schematic diagram of the mechanism of localized corrosion of Cr-added steel under wet/dry conditions.

The Cr alloying element accelerates localized corrosion under Cl-containing wet/dry conditions unlike the immersion condition. The mechanism of localized corrosion of Cr-added steel under wet/dry conditions is as follows, and a schematic diagram is shown in Figure 11.

Corrosion of steel begins in areas where the inherent oxide film is weak. During the wet stage, Cl^−^ ions existing in the aqueous adsorption layer move to these weak areas, and a Cl-concentrated region (nest) is formed [32]. The Cl^−^ is adsorbed on the steel surface, and then atmospheric corrosion initiates. Thereafter, Fe^2+^ reacts with H_2_O to form Fe(OH)_2_, and with salt or Cl^−^ in the air to form FeCl_2_.

Fe → Fe^2 +^ + 2e^−^ (3)

Fe^2+^ + 2H_2_O → Fe(OH)_2_ + 2H^+^ (4)

Fe^2+^ + 2Cl^−^ → FeCl_2_ (5)

During the dry stage, Fe(OH)_2_ is transformed into lepidocrocite, and FeCl_2_ formed in the Cl-concentrated region is transformed into akaganeite. After that, lepidocrocite and akaganeite are reduced to amorphous oxide or magnetite in the wet stage. Next, magnetite is re-oxidized into lepidocrocite.

2γ-FeOOH + Fe^2+^ → Fe_3_O_4_ + 2H^+^ (6)

Fe_3_O_4_ + 3/2O_2_ + H_2_O → 3γ-FeOOH (7)

In the atmospheric rusting process, lepidocrocite on the steel surface transforms into amorphous ferric hydroxide, then it converts to goethite. Cl^−^ may facilitate this reaction and promote goethite formation [33,34,35].

γ-FeOOH → FeO_x_(OH)_3−2x_ (amorphous ferric oxyhydroxide) → α-FeOOH (8)

γ-FeOOH → FeO_x_(OH)_2−2x_Cl → α-FeOOH + HCl (9)

Cr^3+^ ions dissolved in the early stages of the corrosion process are more easily deposited as hydroxides near the steel surface compared to the Fe^2+^ ions since the solubility of Fe^2+^ ions is higher than that of Cr^3+^. Additionally, the Cr^3+^ ions act as nuclei for the growth of Cr-goethite. Finally, an ultrafine Cr-goethite layer is formed in the inner rust layer when the wet/dry process is repeated [6]. Since Cr-goethite has cation selectivity, it suppresses the penetration of aggressive anions and improves corrosion resistance. Then, after the formation of Cr-goethite, the inflow of extra Cl^−^ from the outside is blocked, so that Cl^−^ is locally accumulated underneath the Cr-enriched layer.

During the wet stage, Cl^−^ ions in the akaganeite formed in the inner rust layer are dissolved and eluted in water, resulting in the formation of FeCl_2_ and CrCl_3_. The hydrolysis reactions of these Fe and Cr salts occur.

FeCl_2_ + 2H_2_O → Fe(OH)_2_ + 2HCl (10)

CrCl_3_ + 3H_2_O → Cr(OH)_3_ + 3HCl (11)

The pH of the steel surface is reduced by the hydrolysis reaction of Fe and Cr [36,37]. Since Cr tends to strongly hydrolyze up to pH 1.4, it enhances the susceptibility to localized corrosion compared to the hydrolysis reaction of Fe [38]. That is, FeCl_2_ and CrCl_3_ are formed from the Cl^-^ nest developed in the early stage, and the CrCl_3_-formed regions are locally accelerated. Even if Cr is not added, Cl^−^ may cause localized corrosion. However, when Cr is added, localized corrosion is more accelerated since Cr is strongly hydrolyzed to a very low pH.

Finally, when the wet/dry cycle is continuously repeated, the rust layer is exfoliated and loses its protective property. Then, Cl^−^ easily penetrates into the gap between the separated rust layer and substrate, which accelerates the localized corrosion of ACS.

## 4. Conclusions

In this study, the effect of Cr alloying element on the corrosion properties of ACS in aqueous and atmospheric conditions was investigated using electrochemical measurements and a CCT. The conclusions based on the investigations are as follows:

- In the electrochemical measurement results, the Cr alloying element improves the corrosion resistance of the ACS that was immersed in the Cl-containing aqueous solution.
- Cl is concentrated at the metal/rust interface in all of the specimens regardless of Cr content after the CCT. The Cl is uniformly concentrated and distributed on the 0 Cr steel, whereas Cl is localized and non-uniformly concentrated on the Cr-added steels. The PF of the Cr-added steels is higher than that of the 0 Cr steel during the CCT.
- The inner rust layer consists of Cl-containing akaganeite and Cr-goethite, while the outer rust layer is composed of amorphous iron oxyhydroxide mixed with various types of rust.
- FeCl_2_ and CrCl_3_ are formed from the Cl nest developed in the early stage, and the pitting at CrCl_3_-formed regions is locally accelerated because Cr is strongly hydrolyzed to a very low pH.

## Figures and Tables

**Table 1:** Chemical compositions of the specimens (unit: wt.%).

Steels	Cr	C	Si	Mn	Fe
0 Cr	0.01	0.10	0.52	2.49	Bal.
0.3 Cr	0.32	0.10	0.52	2.49	Bal.
0.5 Cr	0.50	0.10	0.52	2.49	Bal.

**Table 2:** Potentiodynamic polarization test results in 3.5 wt.% NaCl solution.

Parameter	0 Cr	0.3 Cr	0.5 Cr
E_corr_ (mV_SCE_)	−686.5 ± 1.4	−683.6 ± 5.4	−693.2 ± 12.6
I_corr_ (μA/cm^2^)	21.3	20.2	17.4

**Table 3:** Parameters from electrochemical impedance spectroscopy measurements.

Steel	Immersion Time	R_s_ (Ω·cm^−2^)	CPE1		R_film_ (Ω·cm^−2^)	CPE2		R_ct_ (Ω·cm^−2^)	R_p_ (Ω·cm^−2^)
			Q_film_ (Ω^−1^ cm^−^^2^·s^n^)	*n*_1_		Q_ct_ (Ω^−1^· cm^−^^2^·s^n^)	*n*_1_		
0 Cr	0 h	1.424	7.52 × 10^−^^4^	0.8355	29.9	3.97 × 10^−^^4^	0.9671	636.5	665.7
	1 h	2.531	6.11 × 10^−^^4^	0.8146	192.6	1.29 × 10^−^^4^	0.9516	951.6	1144.2
	2 h	2.522	6.36 × 10^−^^4^	0.8037	294	1.48 × 10^−^^4^	0.9883	801.2	1095.2
	3 h	2.542	6.65 × 10^−^^4^	0.801	239.9	1.55 × 10^−^^4^	0.9724	831.3	1071.2
	4 h	2.549	6.84 × 10^−^^4^	0.796	267.4	1.61 × 10^−^^4^	0.997	752.1	1019.5
	5 h	2.574	6.72 × 10^−^^4^	0.7959	217.5	1.73 × 10^−^^4^	0.9593	766.3	983.8
	6 h	2.589	6.81 × 10^−^^4^	0.7953	203.7	1.85 × 10^−^^4^	0.9509	733.1	936.8
0.3 Cr	0 h	1.773	1.71 × 10^−^^4^	1	3	9.06 × 10^−^^4^	0.7621	1304	1307
	1 h	1.227	2.05 × 10^−^^4^	0.9653	32.3	3.76 × 10^−^^4^	0.7516	1210	1242.3
	2 h	1.22	1.92 × 10^−^^4^	0.9714	29.2	3.79 × 10^−^^4^	0.7541	1151	1180.2
	3 h	1.212	1.83 × 10^−^^4^	0.9759	26.5	3.90 × 10^−^^4^	0.7497	1215	1241.5
	4 h	1.212	1.69 × 10^−^^4^	0.9843	24.3	4.00 × 10^−^^4^	0.7475	1223	1247.3
	5 h	1.217	1.59 × 10^−^^4^	0.9914	23.7	4.10 × 10^−^^4^	0.7442	1266	1289.7
	6 h	1.215	1.57 × 10^−^^4^	0.993	23.1	4.08 × 10^−^^4^	0.7424	1285	1308.1
0.5 Cr	0 h	2.809	4.71 × 10^−^^4^	0.8449	21.6	2.14 × 10^−^^4^	0.8069	1414	1435.6
	1 h	1.986	5.09 × 10^−^^4^	0.8474	59	1.63 × 10^−^^4^	0.7983	1543	1602
	2 h	1.978	5.18 × 10^−^^4^	0.8405	63.5	1.67 × 10^−^^4^	0.7818	1419	1482.5
	3 h	1.964	3.86 × 10^−^^4^	0.8637	20.6	2.95 × 10^−^^4^	0.7261	1270	1290.6
	4 h	1.963	3.24 × 10^−^^4^	0.8782	14.9	3.39 × 10^−^^4^	0.7095	1489	1503.9
	5 h	2.001	1.18 × 10^−^^4^	0.9798	4.5	5.31 × 10^−^^4^	0.7418	1502	1506.5
	6 h	2.013	9.42 × 10^−^^4^	0.9999	3.6	5.44 × 10^−^^4^	0.748	1542	1545.6
